# Multifunctional selenium nanoparticles with Galangin-induced HepG2 cell apoptosis through p38 and AKT signalling pathway

**DOI:** 10.1098/rsos.180509

**Published:** 2018-11-28

**Authors:** Yinghua Li, Min Guo, Zhengfang Lin, Mingqi Zhao, Yu Xia, Changbing Wang, Tiantian Xu, Bing Zhu

**Affiliations:** Center Laboratory, Guangzhou Women and Children's Medical Center, Guangzhou Medical University, Guangzhou 510120, People's Republic of China

**Keywords:** selenium nanoparticles, Galangin, caspase-3, reactive oxygen species, apoptosis

## Abstract

The morbidity and mortality of hepatocellular carcinoma, the most common cancer, are increasing continuously worldwide. Galangin (Ga) has been demonstrated to possess anti-cancer effect, but the efficacy of Ga was limited by its low permeability and poor solubility. To develop aqueous formulation and improve the anti-cancer activity of Ga, surface decoration of functionalized selenium nanoparticles with Ga (Se@Ga) was synthesized in the present study. The aim of this study was to evaluate the anti-cancer effect of Se@Ga and the mechanism on HepG2 cells. Se@Ga-induced HepG2 cell apoptosis was confirmed by depletion of mitochondrial membrane potential, translocation of phosphatidylserine and caspase-3 activation. Furthermore, Se@Ga enhanced the anti-cancer activity of HepG2 cells through ROS-mediated AKT and p38 signalling pathways. In summary, these results suggest that Se@Ga might be potential candidate chemotherapy for cancer.

## 1. Background

Hepatocellular carcinoma (HCC) is considered as one of the most common malignancies and ranks third in cancer-associated deaths around the world. [1,2]. However, the early stage diagnosis of HCC is difficult and the prognosis is less satisfactory [3,4]. Moreover, HCC is metastatic or advanced at the time of diagnosis, and the local therapies are unsuitable [5,6]. Furthermore, due to the insensitivity of HCC to chemotherapy and high metastatic potential, the survival of patients with HCC is very difficult [7]. Finally, chemotherapy drugs possess potential drawbacks, such as short half-life, poor aqueous solubility and significant toxicity [8]. Therefore, it remains an urgent medical need to discover new systemic chemotherapy for HCC.

Galangin (3,5,7-trihdroxyflavon) is a natural compound extracted from the *Alpinia galanga* root with high concentration levels [9]. Galangin exhibits suppressive role in various tumour cells, including anti-cancer and anti-inflammation through various signalling pathways [10]. Galangin exerts its anti-proliferative effect in the progression of various cancer cells [11]. But the anti-cancer efficacy is limited by its low permeability and poor water solubility, the molecular mechanisms are not enough understood.

Recently, nanobiotechnology, a breakthrough technology, is provided for anti-cancer therapy [12,13]. It improves the bioavailability and solubility of anti-cancer drugs, reducing side effects of drugs, strengthening molecular targeting and other biomedical applications [14,15]. Nanomaterials are promising nanocarriers with the peculiar properties including high stability, thermal properties, controllable morphology, soluble behaviours and surface functionalization, and are alternatives for traditional medicines [16,17]. Of them, selenium nanoparticles (SeNPs) attract much attention with the unique antimicrobial activities [18,19]. Selenium is an essential nutritional trace element with the regulation of cellular redox homeostasis [20,21]. It is an integral component of several selenoproteins which control several crucial biological processes, such as reactive oxygen species (ROS) elimination [19,22]. ROS play a key role in physiological processes on apoptosis [23,24]. ROS are generated in several cellular systems, such as plasma membrane, cytosol, peroxisomes, endoplasmic reticulum and mitochondria [25,26]. The imbalance of ROS generation could trigger oxidative stress which is related to much pathology, including cancer and other diseases [27]. We hypothesize to design Ga-functionalized SeNPs (Se@Ga) to enhance the cure rate of HCC.

## 2. Material and methods

### 2.1. Materials

The HepG2 cells were obtained from ATCC^®^, CCL-136^™^. LO2 cells (normal human liver cell line) were provided from Cells Bank of the Chinese Academy of Sciences (Shanghai). DMEM and FBS were purchased from Gibco. Na_2_SeO_3_, Vitamin C, PI, DCF-DA and MTT were all obtained from Sigma. Caspase-3 (#9662), AKT (#9272), T-p38 (#9212) and β-actin antibody (#3700) were purchased from CST.

### 2.2. Preparation and characterization of Se@Ga

Se@Ga nanoparticles were synthesized as follows: briefly, 0.25 ml of stock solution (0.1 M) of Na_2_SeO_3_ was gradually added into 2 ml stock solution (50 mM) of Vitamin C. Then, 2 µl of Ga solution (44 mM) was added into the SeNP solution. The Se@Ga complex was purified overnight by dialysis. Se@Ga nanoparticles were sonicated and then filtered through 0.2 µm pore size. They were characterized by various methods. The concentration of SeNPs was measured by ICP-AES. Se@Ga nanoparticle samples were prepared by dispersing the particles onto a holey carbon film on copper grids. The micrographs were obtained for TEM operated at an accelerating voltage of 80 kV. EDX analysis was carried out on an EX-250 system to examine the elemental composition of Se@Ga. FT-IR samples were recorded on an Equinox 55 IR spectrometer (in the range of 4000–500 cm^−1^) using the KBr-disc method. The particle size distribution and zeta potential were determined by Zetasizer Nano ZS particle analyser.

### 2.3. Cell culture and viability assay

The cell proliferative inhibition by Se@Ga nanoparticles was measured, as previously described [28]. Briefly, the cells were incubated with SeNPs, Galangin and Se@Ga at a density of 4 × 10^4^ cells for 24 h. Then, 20 µl of MTT solution was added to each well and incubated for 5 h [29]. The cell viability was determined by detecting the percentage of MTT reduction relative to the absorbance of control. Synergy was evaluated by the calculation of *in vitro* fractional inhibitory concentration-index values: minimum inhibitory concentration (MIC) of drug A combination present in Se@Ga of Se; MIC of drug B combination present in Se@Ga of Ga; MIC of drug A alone corresponded to free SeNPs; MIC of drug B alone corresponded to free Ga. Fractional IC (FIC) was calculated as follows: (MIC drug A combination/MIC drug A alone) + (MIC drug B combination/MIC drug B alone). FIC was 0.375, below 0.5, indicating synergy. In this study, the FIC index was basically interpreted as follows: FIC < 0.5, synergy; FIC between 0.5 and 2, indifference; FIC > 2, antagonism.

### 2.4. Scratch assay

The anti-cell migration effect of Se@Ga on HepG2 cells was detected by a scratch assay [30]. In brief, after the cell confluence reached 80%, cell monolayers were wounded with a sterile microtip and washed with PBS to discard detached cells. Then, the cells were treated with Se, Ga, Se@Ga and incubated for 24 h. After that, the wound closure was observed and photographed by an Olympus microscope at 0 and 24 h.

### 2.5. Mitochondrial membrane potential measurement (Δ*Ψ*_m_)

JC-1 was used to detect the mitochondrial membrane potential by Se@Ga in HepG2 cells, as previously reported [31]. The cells cultured in six-well plates were released by trypsinization, resuspended in PBS buffer with 10 µg ml^−1^ JC-1 and then incubated at 37°C for 30 min. The cells were then harvested by centrifugation, resuspended in PBS and analysed by flow cytometry. JC-1 fluorescence was measured with excitation (485 nm) and dual emission (shift from green at 530 nm to red at 590 nm).

### 2.6. Annexin-V/PI double-staining assay

Translocation of phosphatidylserine in HepG2 cells treated with Se@Ga was detected, as previously described [32]. In brief, the cells were seeded into six-well plates till 70% confluence and then incubated with SeNPs, Ga and Se@Ga for 24 h. The cells were then washed three times by PBS and stained with Annexin-V/PI for 30 and subjected to flow cytometric analysis.

### 2.7. Caspase-3 activity

Caspase-3 activity was determined by a fluorometric method, as described in our previous paper [33]. Harvested cell pellets were suspended in cell lysis buffer and incubated on ice for 1 h. After centrifugation at 11 000 × *g* for 30 min, supernatants were collected and immediately measured for protein concentration and caspase activity. For determination of caspase activity, cell lysates were added in 96-well plates and then incubated with specific caspase-3 substrates for 1 h at 37°C. Caspase-3 activity was determined by fluorescence intensity with an excitation of 380 nm and an emission of 460 nm.

### 2.8. Transmission electron microscopic analysis Se@Ga-treated HepG2

Se@Ga-treated HepG2 was negatively stained and morphologically detected by TEM, as previously described [34]. The HepG2 cell was treated with Se@Ga at various time points and then was attached to the carbon-coated collodion grid for 10 min. The grids were stained with 2% phosphotungstic acid in Sorensen phosphate buffer for 2 min. The grids were examined by TEM after rinsing and air-drying the slides.

### 2.9. Determination of ROS generation

ROS accumulation induced by Se@Ga was estimated, as previously described [35]. In brief, HepG2 cells were harvested and suspended in PBS containing 10 µM of DCFH-DA for 30 min. ROS lever was determined by measuring the fluorescence intensity using a microplate reader. ROS generation was indicated by green florescence which was measured with an excitation of 488 nm and an emission of 525 nm. Experiments were performed in triplicate.

### 2.10. Western blotting analysis

Western blotting was performed, as previously reported [36]. Briefly, total intracellular proteins in HepG2 cells treated with Se@Ga were extracted. The protein concentration was examined by the bicinchoninic acid assay. An equal amount of protein was electrophoresed in 12% tricine gels and blocked with 5% non-fat milk in Tris-buffered saline Tween-20 buffer for 1 h. The membranes were incubated with primary antibodies at 1 : 1000 dilutions overnight at 4°C with continuous agitation. Then, the membranes were incubated with secondary antibody conjugates with horseradish peroxides at 1 : 1000 dilutions for 2 h at room temperature, followed by washing three times with Tris-buffered saline Tween-20 buffer. The proteins were visualized on the X-ray film. The densitometry analysis of band intensity was detected by ImageJ.

### 2.11. Statistical analysis

All data were processed using the SPSS 19.0 software. The difference between three and more groups was analysed by one-way ANOVA multiple comparisons. Differences between two groups were evaluated by two-tailed Student's *t-*test. A probability of *p* < 0.05 (*) or *p* < 0.01 (**) indicates statistically significant values.

## 3. Results and discussion

### 3.1. Preparation and characterization of Se@Ga

**Scheme 1.**
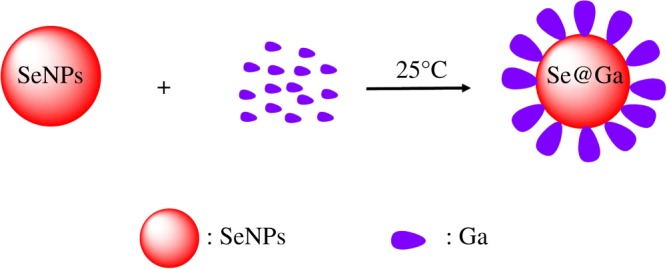
Synthetic route towards Se@Ga.

**Figure 1.**
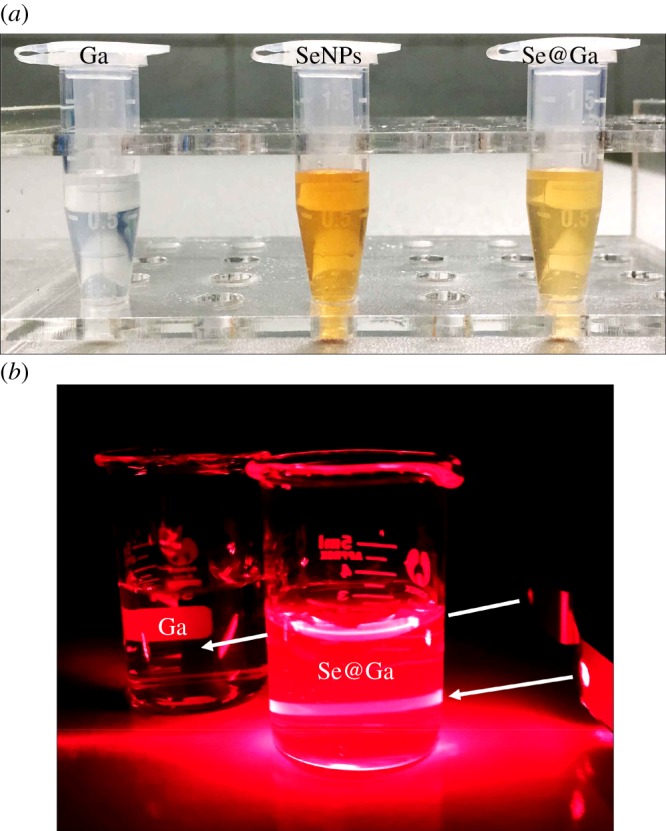
Light image of Ga, SeNPs and Se@Ga. (*a*) The colour change of Ga, SeNPs and Se@Ga. (*b*) Tyndall effect of Se@Ga.

**Figure 2.**
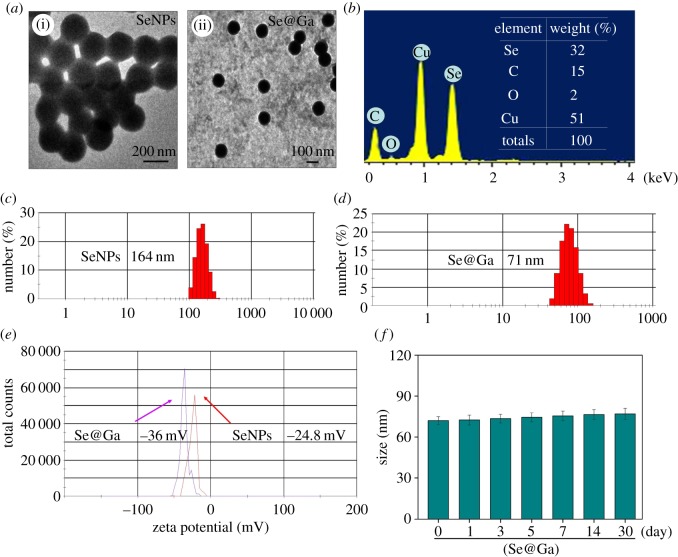
Characterization of SeNPs and Se@Ga. (*a*) TEM image of SeNPs and Se@Ga. (*b*) EDX analysis of Se@Ga. (*c,d*) Size distribution of SeNPs and Se@Ga. (*e*) Zeta potentials of SeNPs and Se@Ga. (*f*) Stability of Se@Ga in aqueous solutions.

**Figure 3.**
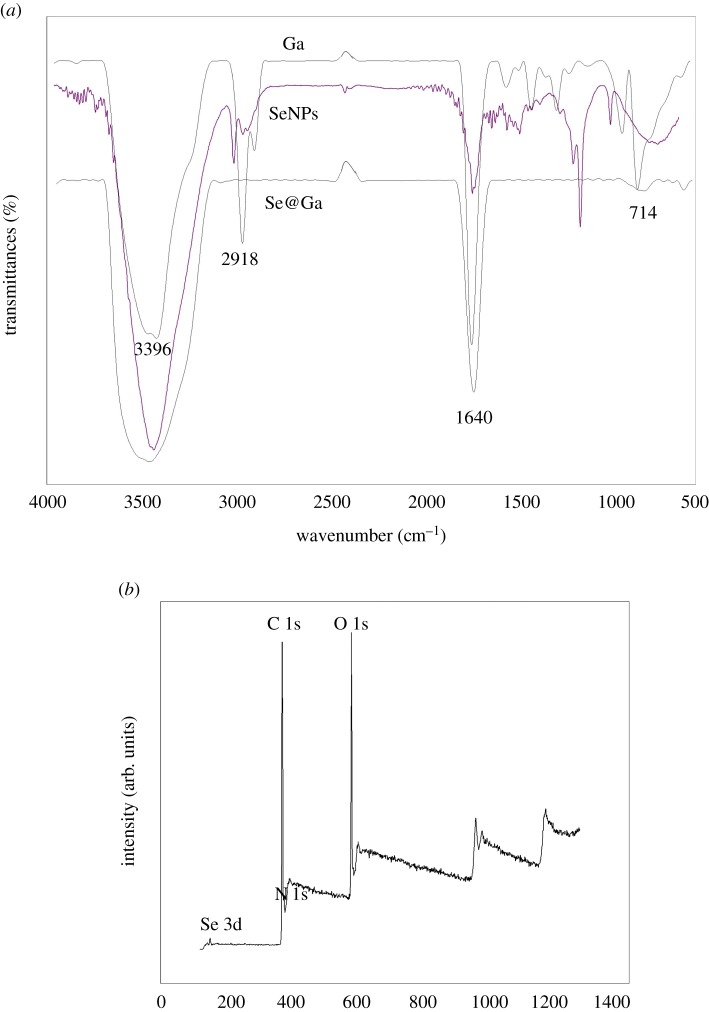
The FT-IR spectra and XPS of Se@Ga. All IR spectra were acquired in the form of KBr plates. (*a*) FT-IR spectrums. (*b*) XPS detection.

Galangin-modified SeNPs (Se@Ga) enhanced anti-cancer effect (scheme 1). Light image of Ga, SeNPs and Se@Ga is shown in figure 1*a*. Owing to the modified of SeNPs, the colour of SeNPs was deeper than that of Se@Ga. As shown in figure 1*b*, the Tyndall effect of Se@Ga indicated that Se@Ga nanoparticles were synthesized. TEM images showed that Se@Ga presented spherical and monodisperse particle (figure 2*a*). As shown in figure 2*b*, EDX indicated the signal of C (15%), O (2%) than from Ga, Se atoms were 32% and Cu (51%) comes from copper grids. Compared with SeNPs (164 nm), Se@Ga presented high uniformity with a minimum diameter of 71 nm (figure 2*c,d*). Ga may reduce the surface-free energy, and the size was lower than that of SeNP. The zeta potential of SeNPs was −24.8 mV and decreased to −36 mV after capping with Galangin (figure 2*e*), which indicated that Se@Ga with positive charge was easier to cross into the cell membrane. Furthermore, size distribution of Se@Ga revealed that the decorated SeNPs were stable at least for 30 days (figure 2*f*), which also indicated that Se@Ga was highly stable in aqueous solutions. FT-IR spectra of Ga, SeNPs and Se@Ga are shown in figure 3*a*. Ga displays IR absorbance peaks at 3396, 2918, 1640 and 714 cm^−1^ corresponding to –CH_3_, –CH_2_, C=O and –C-H, respectively. The absence of these peaks in Se@Ga indicated the formation of Se@Ga. As shown in figure 3*b*, the C 1s and O 1s peak in the spectrum of Se@Ga further confirmed that Ga has been successfully conjugated to the SeNPs, and IR and XPS support the formation of Se–O bond in Se@Ga.

### 3.2. *In vitro* cytoxicity of Se@Ga

**Figure 4.**
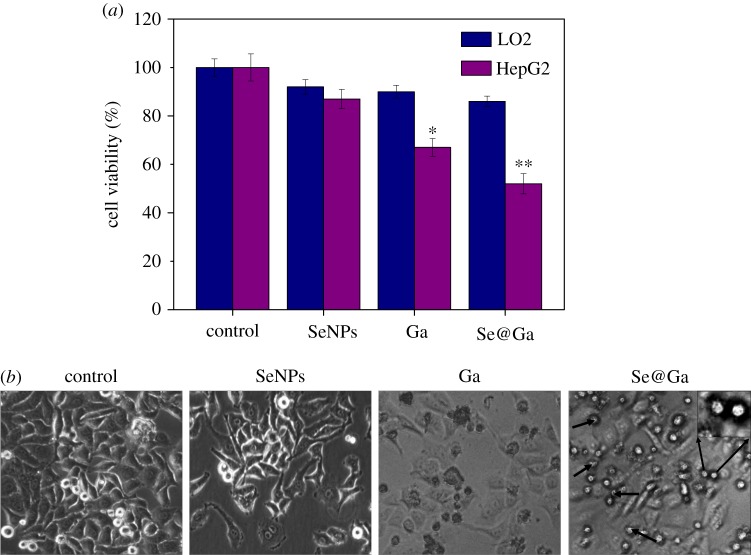
Cytotoxic effects were monitored by Se@Ga on HepG2 cells. (*a*) Cell viability of Se@Ga-treated HepG2 cells and normal cells was determined by the MTT assay. The cells were treated with SeNPs, Ga and Se@Ga for 24 h. (*b*) After treatment with Se@Ga, the morphological changes of HepG2 cells included cell number reduction with cell rounding, cell vacuoles and cytoplasm shrinkage.

The inhibition of HepG2 cell proliferation by SeNPs, Galangin and Se@Ga was measured through the MTT assay. As shown in figure 4*a*, the cell viability of HepG2 was dramatic lower than LO2 cells. The cell viability of HepG2 by SeNPs and Ga was 87% and 67%, respectively, but when treated with Se@Ga, the cell viability decreased to 52%. Compared with SeNPs and Galangin, Se@Ga significantly inhibited the growth of HepG2 cells. As shown in figure 4*b*, the effects of SeNPs, Galangin and Se@Ga on the growth of HepG2 cells were further confirmed. After treating with Se@Ga, the cell numbers reduced with cytoplasm shrinkage. Synergy was evaluated by the calculation of *in vitro* fractional inhibitory concentration-index values: MIC of drug A combination present in Se@Ga of Se (125 µM); MIC of drug B combination present in Se@Ga of Ga (11 µM); MIC of drug A alone corresponded to free SeNPs (1 mM); MIC of drug B alone corresponded to free Ga (44 µM). FIC was calculated as (MIC drug A combination/MIC drug A alone) + (MIC drug B combination/MIC drug B alone) = 125 µM/1 mM + 11 µM /44 µM = 0.375. FIC was 0.375, below 0.5, indicating synergy. In this study, the FIC index was basically interpreted as follows: FIC < 0.5, synergy; FIC between 0.5 and 2, indifference; FIC > 2, antagonism. The results suggest that Se@Ga effectively inhibited the proliferation of HepG2.

### 3.3. Depletion of mitochondrial membrane potential (Δ*Ψ*_m_) and translocation of phosphatidylserine induced by Se@Ga

**Figure 5.**
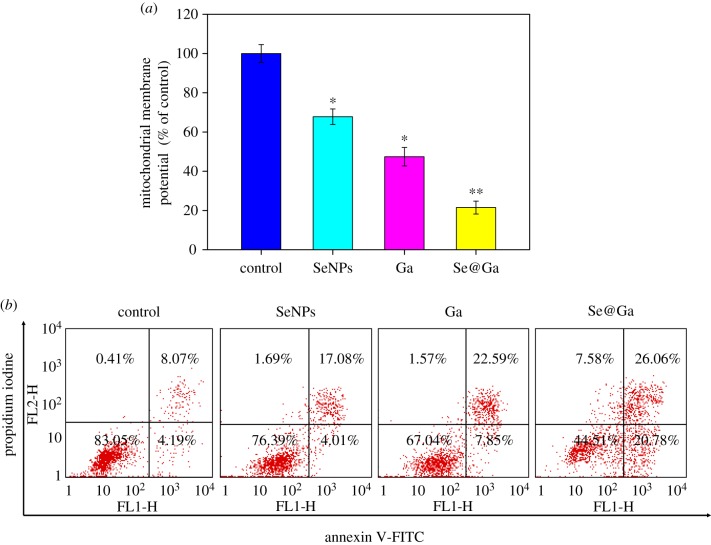
Depletion of mitochondrial membrane potential and translocation of phosphatidylserine induced by SeNPs, Ga and Se@Ga. (*a*) Mitochondrial membrane potential of HepG2 cells exposed to SeNPs, Ga and Se@Ga. (*b*) Translocation of phosphatidylserine induced by SeNPs, Ga and Se@Ga in HepG2 cells.

As shown in figure 5*a*, the mitochondrial membrane potentials of SeNPs, Ga and Se@Ga reduced significantly to 67.8, 47.4 and 21.5%, respectively. These results revealed that Se@Ga triggered HepG2 apoptotic cells by induction of mitochondrial dysfunction. As shown in figure 5*b*, Dot plot results of HepG2 cell-treated groups showed the presence of both early and late apoptotic cells. HepG2 cells treated with Se@Ga revealed the increased cell number of apoptosis.

### 3.4. Induction of caspase cleavage by Se@Ga

**Figure 6.**
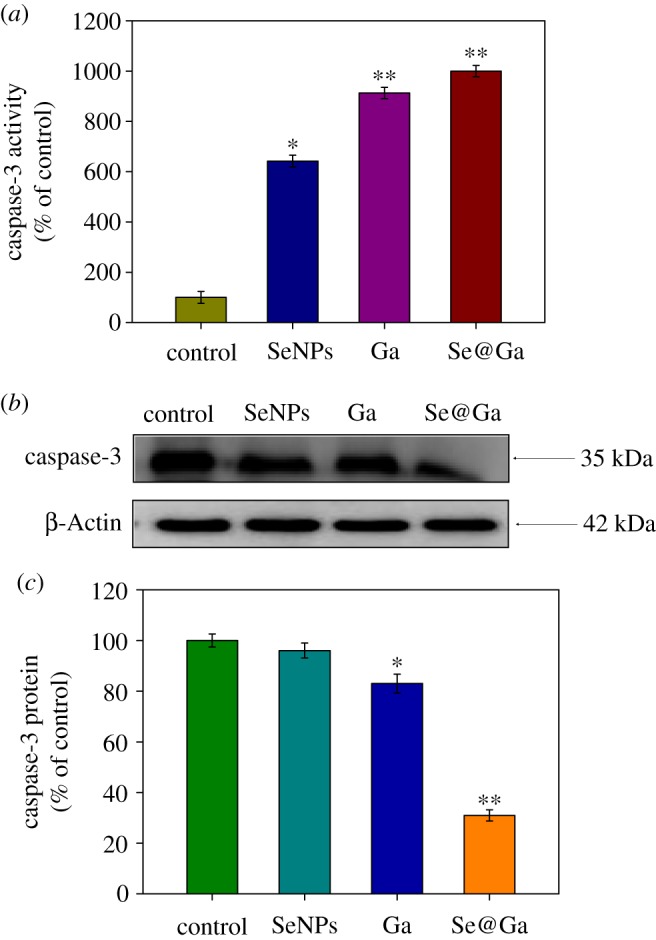
Se@Ga-induced caspase-3 activation. (*a*) HepG2 cells were treated with different treatments for 24 h and activation of caspase-3 was measured. (*b,c*) The expression level of caspase-3 protein was measured by Western blotting.

The caspase family of aspartate-specific cysteine proteases plays important roles in the initiation and execution of apoptosis. As shown in figure 6*a*, compared with the control group (100%), SeNPs (642%) and Galangin (913%), treatments of HepG2 cells with Se@Ga (1000%) significantly increased the activity of caspase-3. Meanwhile, to determine whether caspase family was activated in HepG2 cells exposed to Se@Ga, the activities of caspase 3 were measured by Western blotting, as shown in figure 6*b,c*. The protein expression level of caspase-3 (control 100%, SeNPs 96%, Ga 83% and Se@Ga 31%) was downregulated with different treatments. The results show that Se@Ga significantly strengthened the activation of caspase-3 and induced the HepG2 cell apoptosis.

### 3.5. Se@Ga inhibited the migration of HepG2 cells and TEM image of thin sections

**Figure 7.**
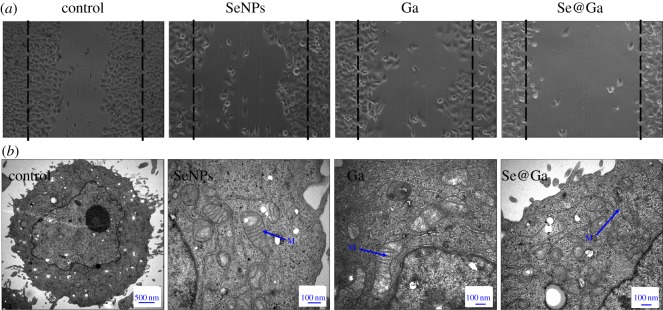
(*a*) Wound-healing assay was conducted to analyse cell migration. The migration of HepG2 cells was dramatically suppressed after treatment with Se@Ga for 24 h. (*b*) TEM images of thin sections of HegG2 cells treated with different groups.

As presented in figure 7*a*, the HepG2 cells were exposed to SeNPs, Ga and Se@Ga, and the cellular migration was analysed by a scratch assay. In the control group, the wounded gap was almost completely occupied by the migrating cells after 24 h, while in the Se@Ga-treated group, this gap was not occupied by migrating cells. This result suggested that Se@Ga significantly inhibited the migration ability of HepG2 cells. The morphology change of HepG2 cells treated with Se@Ga was observed by TEM. As shown in figure 7*b*, when incubated with Se@Ga, TEM image indicates few cells with the disappearance of microvilli, a shrinking cytoplasm, distorted organelles and condensed chromatin. The percentage of cells that lost adhesion and shrunk was decreased after treatment with Se@Ga.

### 3.6. Induction of ROS generation by Se@Ga

**Figure 8.**
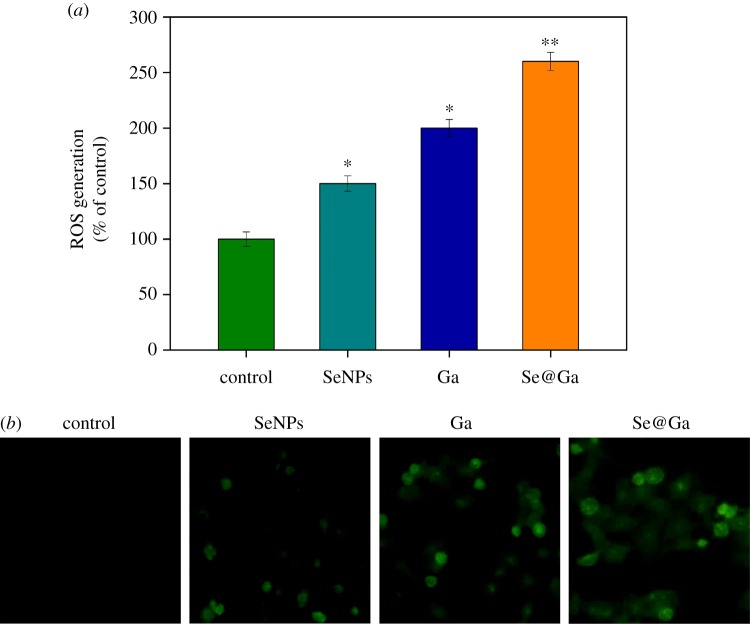
The intracellular ROS of HepG2 cells exposed with Se@Ga. (*a*) ROS overproduction induced by Se@Ga was measured by a fluorescence plate reader. (*b*) Representative fluorescence images of cells stained by DCFH-DA.

ROS generation was determined by the DCF fluorescence assay to reveal its role in the action mechanisms of Se@Ga. As shown in figure 8*a* (control 100%, SeNPs 150%, Ga 200% and Se@Ga 260%), the ROS generation of HepG2 cells increased significantly after treatment with Se@Ga. As shown in figure 8*b*, the fluorescent intensity of DCF in HepG2 cell exposure to Se@Ga was the most strongest in treatment groups. The results indicate the involvement of ROS in the anti-cancer action of Se@Ga.

### 3.7. Activation of ROS-mediated signalling pathways by Se@Ga

**Figure 9.**
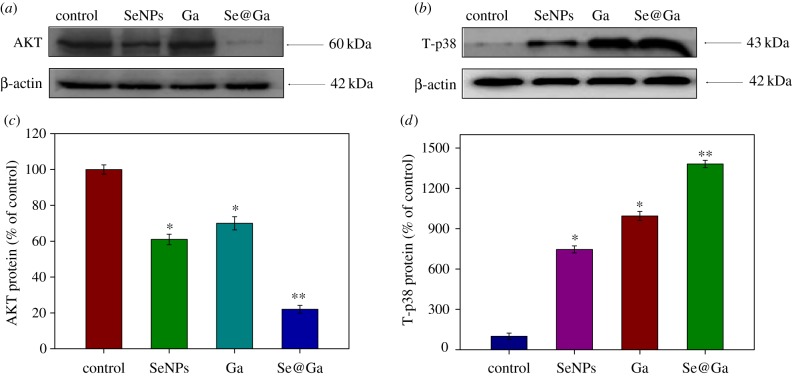
Activation of ROS-mediated apoptosis signal pathways by Se@Ga in HepG2 cells. (*a,c*) Activation of AKT signalling pathway. (*b,d*) Activation of p38 signal pathway.

Intracellular ROS overproduction could trigger DNA damage and cause a series of different signalling pathways, such as AKT and MAPK signalling pathways. Western blot analysis was used to examine the effects of Se@Ga on the expression of AKT and p38. As shown in figure 9*a,c*, the expression of total AKT was downregulated after treatment with Se@Ga (control 100%, SeNPs 61%, Ga 70% and Se@Ga 22%). Meanwhile, as shown in figure 9*b,d*, HepG2 cells treated with Se@Ga effectively increased the expression of total p38 in HepG2 cells (control 100%, SeNPs 746%, Ga 995% and Se@Ga 1382%). The dates suggest that MAPK pathways were involved in cancer cell apoptosis induced by Se@Ga. The results reveal that nanosystem induces HepG2 cells apoptosis through regulation of ROS-mediated AKT and p38 signalling pathways.

## 4. Conclusion

Galangin-modified SeNPs were successfully fabricated in the study. Se@Ga enhanced the drug sensitivity and induced apoptosis of cancer cells rather than normal cells. The underlying molecular mechanisms indicated that Se@Ga activated caspase-3-mediated HepG2 cell apoptosis via ROS generation. Furthermore, our results indicated the apoptotic signalling pathway through ROS-mediated triggered by the Se@Ga in HepG2 cells, including AKT and p38 signalling pathways. Taken together, for achieving anti-cancer activity, the strategy to use SeNPs as a carrier of Ga could be a highly efficient way. Se@Ga may be a candidate for the next assessment as chemotherapeutics of cancers, especially HCC.

## Data accessibility

We have conducted our experiments systematically and reported their experimental procedure clearly in the experimental section and provided all necessary data in the results and discussion section in the main manuscript.

## References

[1] Li YH, Guo M, Lin ZF, Zhao MQ, Xiao MS, Wang C, Xu T, Chen T, Zhu B 2016 Polyethylenimine-functionalized silver nanoparticle-based co-delivery of paclitaxel to induce HepG2 cell apoptosis. Int. J. Nanomed. 11, 6693–6702. (10.2147/IJN.S122666) PMC5154725 27994465

[2] Dong Y, Zou JJ, Su S, Huang HY, Deng YZ, Wang BR, Li W 2015 MicroRNA-218 and microRNA-520a inhibit cell proliferation by downregulating E2F2 in hepatocellular carcinoma. Mol. Med. Rep. 12, 1016–1022. (10.3892/mmr.2015.3516) 25816091 PMC4438929

[3] Zhu B, Li YH, Lin ZF, Zhao MQ, Xu TT, Wang CB, Deng N 2016 Silver nanoparticles induce HePG-2 cells apoptosis through ROS-mediated signaling pathways. Nanoscale Res. Lett. 11, 198–205. (10.1186/s11671-016-1419-4) 27075340 PMC4830774

[4] Gong XL, Qin SK 2016 Progress in systemic therapy of advanced hepatocellular carcinoma. World J. Gastroenterol. 22, 6582–6594. (10.3748/wjg.v22.i29.6582) 27547002 PMC4970483

[5] Ling D, Xia H, Park W, Hackett MJ, Song C, Na K, Hui KM, Hyeon T 2014 pH-sensitive nanoformulated triptolide as a targeted therapeutic strategy for hepatocellular carcinoma. ACS Nano 8, 8027–8039. (10.1021/nn502074x) 25093274

[6] Thapa RK, Choi JY, Poudel BK, Hiep TT, Pathak S, Gupta B, Choi H-G, Yong CS, Kim JO 2015 Multilayer-coated liquid crystalline nanoparticles for effective sorafenib delivery to hepatocellular carcinoma. ACS Appl. Mater. Interfaces 7, 20 360–20 368. (10.1021/acsami.5b06203) 26315487

[7] Rand D, Ortiz V, Liu YA, Derdak Z, Wands JR, Taticek M, Rose-Petruck C 2011 Nanomaterials for X-ray imaging: gold nanoparticle enhancement of X-ray scatter imaging of hepatocellular carcinoma. Nano Lett. 11, 2678–2683. (10.1021/nl200858y) 21644516 PMC3138192

[8] Li JH, Sharkey CC, Huang DT, King MR 2015 Nanobiotechnology for the therapeutic targeting of cancer cells in blood. Cell. Mol. Bioeng. 8, 137–150. (10.1007/s12195-015-0381-z) 25798204 PMC4361771

[9] Song W, Yan CY, Zhou QQ, Zhen LL 2017 Galangin potentiates human breast cancer to apoptosis induced by TRAIL through activating AMPK. Biomed. Pharmacother. 89, 845–856. (10.1016/j.biopha.2017.01.062) 28282786

[10] Wang Y, Lin B, Li H, Lan L, Yu H, Wu S, Wu J, Zhang H 2017 Galangin suppresses hepatocellular carcinoma cell proliferation by reversing the Warburg effect. Biomed. Pharmacother. 95, 1295–1300. (10.1016/j.biopha.2017.09.056) 28938520

[11] Wang HX, Tang C 2017 Galangin suppresses human laryngeal carcinoma via modulation of caspase-3 and AKT signaling pathways. Oncol. Rep. 38, 703–714. (10.3892/or.2017.5767) 28677816 PMC5562077

[12] Li YH, Lin ZF, Guo M, Xia Y, Zhao MQ, Wang CB, Xu T, Chen T, Zhu B 2017 Inhibitory activity of selenium nanoparticles functionalized with oseltamivir on H1N1 influenza virus. Int. J. Nanomed. 12, 5733–5743. (10.2147/IJN.S140939) PMC5557909 28848350

[13] Huang YY, He LZ, Liu W, Fan CD, Zheng WJ, Wong YS, Chen T 2013 Selective cellular uptake and induction of apoptosis of cancer-targeted selenium nanoparticles. Biomaterials 34, 7106–7116. (10.1016/j.biomaterials.2013.04.067) 23800743

[14] Li YH, Lin ZF, Zhao MQ, Xu TT, Wang CB, Hua L, Wang H, Xia H, Zhu B 2016 Silver nanoparticle based codelivery of oseltamivir to inhibit the activity of the H1N1 influenza virus through ROS-mediated signaling pathways. ACS Appl. Mater. Interfaces 8, 24 385–24 393. 10.1021/acsami.6b06613 27588566

[15] Wang YZ, Fan Z, Shao L, Kong XW, Hou XJ, Tian DR, Sun Y, Xiao Y, Yu L 2016 Nanobody-derived nanobiotechnology tool kits for diverse biomedical and biotechnology applications. Int. J. Nanomed. 11, 3287–3302. (10.2147/IJN.S107194) PMC4959585 27499623

[16] Shen S, Sun CY, Du XJ, Li HJ, Liu Y, Xia JX, Zhu Y-H, Wang J 2015 Co-delivery of platinum drug and si*Notch1* with micelleplex for enhanced hepatocellular carcinoma therapy. Biomaterials 70, 71–83. (10.1016/j.biomaterials.2015.08.026) 26302232

[17] Khan M, Khan M, Al-Marri AH, Al-Warthan A, Alkhathlan HZ, Siddiqui MRH, Nayak VL, Kamal A, Adil SF. 2016 Apoptosis inducing ability of silver decorated highly reduced graphene oxide nanocomposites in A549 lung cancer. Int. J. Nanomed. 11, 873–883. 10.2147/IJN.S100903 PMC4788371 27022256

[18] Jamroz E, Kopel P, Juszczak L, Kawecka A, Bytesnikova Z, Milosavljevic V, Kucharek M, Makarewicz M, Adam V 2018 Development and characterization of furcellaran-gelatin films containing SeNPs and AgNPs that have antimicrobial activity. Food Hydrocolloid. 83, 9–16. (10.1016/j.foodhyd.2018.04.028)

[19] Li XL, Ma LJ, Zheng WJ, Chen TF 2014 Inhibition of islet amyloid polypeptide fibril formation by selenium-containing phycocyanin and prevention of beta cell apoptosis. Biomaterials 35, 8596–8604. (10.1016/j.biomaterials.2014.06.056) 25034964

[20] Li YH, Li XL, Zheng WJ, Fan CD, Zhang YB, Chen TF 2013 Functionalized selenium nanoparticles with nephroprotective activity, the important roles of ROS-mediated signaling pathways. J. Biomed. Mater. Res. B Appl. Biomater. 1, 6365–6372. (10.1039/c3tb21168a) 32261335

[21] Yu B, Li XL, Zheng WJ, Feng YX, Wong YS, Chen TF 2014 pH-responsive cancer-targeted selenium nanoparticles: a transformable drug carrier with enhanced theranostic effects. J. Biomed. Mater. Res. B Appl. Biomater. 2, 5409–5418. (10.1039/C4TB00399C) 32261761

[22] Liu W, Li XL, Wong YS, Zheng WJ, Zhang YB, Cao WQ, Chen T 2012 Selenium nanoparticles as a carrier of 5-fluorouracil to achieve anticancer synergism. ACS Nano. 6, 6578–6591. (10.1021/nn202452c) 22823110

[23] Li YH, Lin ZF, Zhao MQ, Guo M, Xu TT, Wang CB, Xia H, Zhu B 2016 Reversal of H1N1 influenza virus-induced apoptosis by silver nanoparticles functionalized with amantadine. RSC Adv. 6, 89 679–89 686. (10.1039/C6RA18493F)

[24] Zhang JX, Wang XL, Vikash V, Ye Q, Wu DD, Liu YL, Dong W 2016 ROS and ROS-mediated cellular signaling. Oxid. Med. Cell Longev. 2016, 1–18. (10.1155/2016/4350965) PMC4779832 26998193

[25] Li YH, Lin ZF, Xu TT, Wang CB, Zhao MQ, Xiao MS, Wang H, Deng N, Zhu B 2017 Delivery of VP1 siRNA to inhibit the EV71 virus using functionalized silver nanoparticles through ROS-mediated signaling pathways. RSC Adv. 7, 1453–1463. (10.1039/C6RA26472G)

[26] Wu HL, Zhu HL, Li XL, Liu ZM, Zheng WJ, Chen TF, Yu B, Wong K-H 2013 Induction of apoptosis and cell cycle arrest in A549 human lung adenocarcinoma cells by surface-capping selenium nanoparticles: an effect enhanced by polysaccharide-protein complexes from *Polyporus rhinocerus*. J. Agric. Food Chem. 61, 9859–9866. (10.1021/jf403564s) 24053442

[27] Lin ZF, Li YH, Guo M, Xu TT, Wang CB, Zhao MQ, Wang H, Chen T, Zhu B 2017 The inhibition of H1N1 influenza virus-induced apoptosis by silver nanoparticles functionalized with zanamivir. RSC Adv. 7, 742–750. (10.1039/C6RA25010F)

[28] Zhang YB, Li XL, Huang Z, Zheng WJ, Fan CD, Chen TF 2013 Enhancement of cell permeabilization apoptosis-inducing activity of selenium nanoparticles by ATP surface decoration. Nanomedicine 9, 74–84. (10.1016/j.nano.2012.04.002) 22542821

[29] Guo M, Li YH, Lin ZF, Zhao MQ, Xiao MS, Wang CB, Xu T, Xia Y, Zhu B 2017 Surface decoration of selenium nanoparticles with curcumin induced HepG2 cell apoptosis through ROS mediated p53 and AKT signaling pathways. RSC Adv. 7, 52 456–52 464. (10.1039/C7RA08796A)

[30] You YY, Hu H, He LZ, Chen TF 2015 Differential effects of polymer-surface decoration on drug delivery, cellular retention, and action mechanisms of functionalized mesoporous silica nanoparticles. Chem-Asian J. 10, 2743–2753. 10.1002/asia.201500769 26248202

[31] Wu HL, Li XL, Liu W, Chen TF, Li YH, Zheng WJ, Man CW-Y, Wong M-K, Wong K-H 2012 Surface decoration of selenium nanoparticles by mushroom polysaccharides-protein complexes to achieve enhanced cellular uptake and antiproliferative activity. J. Mater. Chem. 22, 9602–9610. (10.1039/c2jm16828f)

[32] Feng YX, Su JY, Zhao ZN, Zheng WJ, Wu HL, Zhang YB, Chen T 2014 Differential effects of amino acid surface decoration on the anticancer efficacy of selenium nanoparticles. Dalton Trans. 43, 1854–1861. (10.1039/C3DT52468J) 24257441

[33] Huang YY, Luo Y, Zheng WJ, Chen TF 2014 Rational design of cancer-targeted BSA protein nanoparticles as radiosensitizer to overcome cancer radioresistance. ACS Appl. Mater. Interfaces 6, 19 217–19 228. (10.1021/am505246w) 25314331

[34] Li JM, Wang YY, Zhao MX, Tan CP, Li YQ, Le XY, Ji L-N, Mao Z-W 2012 Multifunctional QD-based co-delivery of siRNA and doxorubicin to HeLa cells for reversal of multidrug resistance and real-time tracking. Biomaterials 33, 2780–2790. (10.1016/j.biomaterials.2011.12.035) 22243797

[35] Lin ZF, Li YH, Guo M, Xiao MS, Wang CB, Zhao MQ, Xu T, Xia Y, Zhu B 2017 Inhibition of H1N1 influenza virus by selenium nanoparticles loaded with zanamivir through p38 and JNK signaling pathways. RSC Adv. 7, 35 290–35 296. (10.1039/C7RA06477B)

[36] Xia Y, Lin ZF, Li YH, Zhao MQ, Wang CB, Guo M, Zhang B, Zhu B 2017 Targeted delivery of siRNA using RGDfC-conjugated functionalized selenium nanoparticles for anticancer therapy. J. Biomed. Mater. Res. B Appl. Biomater. 5, 6941–6952. (10.1039/C7TB01315A) 32264343

